# A randomised controlled trial of antiplatelet therapy in combination with Rt-PA thrombolysis in ischemic stroke: rationale and design of the ARTIS-Trial

**DOI:** 10.1186/1745-6215-11-51

**Published:** 2010-05-12

**Authors:** SM Zinkstok, M Vermeulen, J Stam, RJ de Haan, YB Roos

**Affiliations:** 1 Department of Neurology, Academic Medical Centre, PO Box 22660, 1100 DD, Amsterdam, the Netherlands; 2 Department of Epidemiology and Biostatistics, Academic Medical Centre, PO Box 22660, 1100 DD, Amsterdam, the Netherlands

## Abstract

**Background:**

Thrombolysis with intravenous rt-PA is currently the only approved acute therapy for ischemic stroke. Re-occlusion after initial recanalization occurs in up to 34% in patients treated with rt-PA, probably caused by platelet activation. In acute myocardial infarction, the combination of thrombolysis and antiplatelet therapy leads to a greater reduction of mortality compared to thrombolysis alone. In patients with acute ischemic stroke, several studies showed that patients already on antiplatelet treatment prior to thrombolysis had an equal or even better outcome compared to patients without prior antiplatelet treatment, despite an increased risk of intracerebral bleeding. Based on the fear of intracerebral haemorrhage, current international guidelines recommend postponing antiplatelet therapy until 24 hours after thrombolysis. Remarkably, prior use of antiplatelet therapy is not a contra-indication for thrombolysis. We hypothesize that antiplatelet therapy in combination with rt-PA thrombolysis will improve outcome by enhancing fibrinolysis and preventing re-occlusion.

**Methods/Design:**

ARTIS is a randomised multi-center controlled trial with blind endpoint assessment. Our objective is to investigate whether immediate addition of aspirin to rt-PA thrombolysis improves functional outcome in ischemic stroke. Patients with acute ischemic stroke eligible for rt-PA thrombolysis are randomised to receive 300 mg aspirin within 1.5 hours after start of thrombolysis or standard care, consisting of antiplatelet therapy after 24 hours. Primary outcome is poor functional health at 3 months follow-up (modified Rankin Scale 3 - 6).

**Discussion:**

This is the first clinical trial investigating the combination of rt-PA and acute aspirin by means of a simple and cheap adjustment of current antiplatelet regimen. We expect the net benefit of improved functional outcome will overcome the possible slightly increased risk of intracerebral haemorrhage.

**Trial registration:**

The Netherlands National Trial Register NTR822. The condensed rationale of the ARTIS-Trial has already been published in Cerebrovascular Diseases.

## Background

Stroke is an important cause of death and acquired disability in industrial world. In the large majority of ischemic strokes, cerebral arteries become occluded either by a cardiac embolus or by thrombus formation in atherosclerotic vessel walls. The process of thrombus formation is initiated by two separate but interacting mechanisms: fibrin formation and platelet activation. Current standard treatment in acute ischemic stroke with intravenous recombinant tissue plasminogen activator (rt-PA) aims at breaking down the fibrin cloth in order to restore recanalisation of the occluded artery. Rt-PA treatment results in an absolute 10% reduction of poor outcome compared to placebo [1]. However, benefit from this treatment rapidly declines over time after symptom onset. The number needed to treat to have 1 patient with favourable outcome is 4-5 if treatment is started within 90 minutes after symptom onset as compared to controls [2], while this number increases to 14 if treatment is started between 3 to 4.5 hours after symptom onset [2,3].

Within this current time window, early recanalisation is strongly associated with improved functional outcomes and reduced mortality [4,5]. Unfortunately, recanalisation with intravenous rt-PA is only modest. The overall recanalisation rate observed in 14 pooled intravenous thrombolysis studies was only 43% [5], partly due to re-occlusion. In continuous transcranial Doppler monitoring studies, re-occlusion occurs in 20 - 34% of rt-PA treated patients at a mean time of 65 minutes after start of treatment [6,7]. Moreover, these studies show that re-occlusion accounts for two thirds of the observed clinical deterioration after initial improvement. A recent study confirmed the association between re-occlusion and clinical deterioration and showed that early re-occlusion is highly predictive of long-term poor outcome [8].

Re-occlusion after initial recanalisation is probably initiated by increased platelet activation. Thrombolytic therapy strongly activates the coagulation cascade leading to thrombin formation, a potent platelet activator [9]. This haemostatic activation is maximal at 2 hours after initiation of rt-PA treatment [10]. Exposure of the lipid core of a disintegrating thrombus also leads to increased platelet aggregation. These activated platelets induce secretion of plasminogen activator inhibitor-1, which has been shown to be the responsible factor in t-PA resistance to lysis in platelet rich arterial thrombi [11].

Among all antiplatelet drugs, aspirin (acetylsalicylic acid) is the most widely used drug. Inhibition of platelet aggregation by aspirin is caused by the irreversible acetylation of cyclo-oxygenase 1 and inhibition of prostaglandin thromboxane A2. Aspirin has a rapid onset of action resulting in substantial elimination of activated platelets.

In myocardial infarction, large clinical trials have shown that adding aspirin to thrombolysis prevents re-occlusion thereby improving outcome considerably. The results of the second International Study of Infarct Survival Trial (ISIS-2) showed that mortality was reduced by 42% if patients were treated with streptokinase in combination with aspirin while mortality was reduced by only 25% if patients were treated with streptokinase alone [12]. Aspirin is therefore the standard adjunctive treatment in acute myocardial infarction.

In acute ischemic stroke, the Multicenter Acute Stroke Trial - Italy (MAST-I) duplicated the design of the ISIS 2 and showed an absolute risk reduction of 14% for disability in patients treated with the combination of streptokinase and aspirin as compared streptokinase alone. This overall net benefit overcame an excess mortality rate which was observed in the combination group. Symptomatic intracerebral haemorrhages (SICH) largely contributed to this increased mortality rate [13]. Meta-analysis of the streptokinase trials confirmed a positive effect on functional outcome with concomitant use of aspirin that compensated for higher mortality rates [14]. Besides the high dosage of streptokinase used in these trials, its non-fibrin-selectivity is nowadays generally held responsible for the high number of SICH observed in this study.

The addition of aspirin to rt-PA, which is a fibrin-selective thrombolytic agent, has never been investigated prospectively. In the protocol of the Neurological Institute of Neurological Disorders and Stroke (NINDS) Trial the use of antiplatelet agents was postponed for 24 hours after rt-PA treatment to prevent possible bleeding complications [15]. However, the protocol did allow enrolling patients already on antiplatelet drugs. Current guidelines adopted these trial criteria for fear of SICH [16].

Subgroup analysis of the NINDS-trial reveals that patients with prior aspirin use had a better outcome, with lower frequencies of clinical deterioration and the same SICH rate as compared to patients without previous aspirin use [17]. Regarding the association between clinical deterioration and the occurrence of vessel re-occlusion in ischemic stroke [6,8] and the lower incidence of clinical deterioration in patients with antiplatelet pre-treatment [17], one might suggest that previous antiplatelet therapy prevents re-occlusion. This hypothesis is supported by the observation from recent prospective cohort studies, which confirm this favourable outcome after thrombolysis in patients with prior use of antiplatelet drugs [18,19]. Based on all these observations we hypothesize that immeditate addition of antiplatelet therapy to rt-PA in acute ischemic stroke improves outcome by enhancing clot lysis and preventing re-occlusion after initial recanalisation.

## Methods and Design

### Study design and objective

The Antiplatelet therapy in combination with Recombinant t-PA Thrombolys in Ischemic Stroke (ARTIS) Trial is a multi-center, prospective open, randomised controlled trial with blind endpoint assessment (PROBE-design). We compare direct addition of 300 mg aspirin to intravenous rt-PA thrombolysis for ischemic stroke versus standard thrombolysis care, in which antiplatelet drugs are usually delayed by 24 hours after rt-PA. All participating centres are experienced in thrombolytic treatment for acute stroke.

The primary objective of the ARTIS-Trial is to investigate whether the addition of asprin to standard rt-PA thrombolysis reduces poor outcome in acute ischemic stroke. Poor outcome is defined as death or dependency assessed by the modified Rankin Scale (mRS, score 3-6) at 3 months follow-up.

### Enrolment procedures

The study population are acute ischemic stroke patients who present at participating centres and are treated with intravenous (IV) thrombolysis with rt-PA. Patients aged 18 years or older can be enrolled. Patients will be asked for written informed consent. The trial itself has no other firm exclusion criteria than those established by the judgment of the individual treating physician using local protocols for IV rt-PA treatment. When the patient has a diminished decision-making capacity as result of the stroke (e.g. aphasia), informed consent will be obtained from a representative of the patient. Exclusion of these patients would lead to a selective patient sample. Patients are also excluded if they have:

▪ known antiplatelet therapy in the previous 5 days (in case of uncertainty the patient may be included);

▪ known thrombocytopenia or thrombocyte count < 100 * 10E9/l;

▪ known contra-indications to acetylsalicylic acid treatment;

▪ known anticogualant therapy in the previous 5 days;

▪ known legal incompetence of the patient prior to this stroke.

### Randomisation

Randomisation will be performed per participating centre to ensure a equal distribution of patients between both group. The randomisation procedure will be computer- and web based, using permuted blocks. Randomisation will be stratified by centre, age (≤ 60 years, > 60 years), gender and the time between symptom onset and time of rt-PA bolus (< 2 hours, 2-3 hours, > 3 hours).

### Intervention

In order to prevent delay of start of thrombolytic treatment, informed consent and randomisation procedures will be performed as soon as continuous infusion of rt-PA (0.9 mg/kg) has started after bolus administration (10%) Patients allocated to the active group will receive 300 mg aspirin (Aspégic^®^) as lysine salt intravenous as bolus. Since there is a peak in platelet activation after 2 hours after initiation of rt-PA thrombolysis [10], aspirin will be administered within 1.5 hours after the rt-PA bolus. Patient and treating physician are not blinded for treatment allocation.

We choose to apply aspirin intravenously for two reasons. First, onset of action has to be as soon as possible as re-occlusion starts to occur soon after rt-PA administration [6,7]. Intravenous aspirin leads to faster platelet suppression than oral aspirin, which results in a widely varying uptake [20]. Aspirin may be given simultaneous with the rt-PA continuous infusion, preferably through a different intravenous line. In case of only one intravenous access, rt-PA infusion has to be shortly interrupted in order to administer aspirin through this line with saline flushing before and afterwards.

Second, intravenously administration enables patients having swallowing difficulties caused by their stroke to be included. Exclusion of this subgroup would make the trial prone to inclusion bias.

### Investigational medicinal product

Aspirin intravenous is registered in the Netherlands as Aspégic^® ^(Sanofi-Synthelabo BV). Thrombocyte aggregation is irreversibly reduced by this calcium-ureum-salt, causing longer coagulations times. Aspirin use may lead to gastro-intestinal reactions. However, due to the single use adverse reactions caused by the trial medication are expected to be limited.

Alteplase^® ^(Boehringer Ingelheim GmbH) is essential and important co-medication in the ARTIS-Trial. Interaction of Aspégic^® ^with rt-PA is unknown, although rt-PA treatment might increase the risk of intracerebral bleeding in aspirin pre-treated stroke patients. The characteristics of rt-PA may therefore influence our results even though rt-PA itself is not under investigation.

### Recommendations concerning rt-PA treatment

Patients will receive rt-PA treatment in both groups according to local protocols at participating centres. Recommendations of rt-PA treatment concerning hypertension and thrombocyte count are based on standard international guidelines [16]. Blood pressure should not be lowered with medication prior to rt-PA treatment. If during rt-PA administration blood pressure rises above 180 mmHg systolic or 105 mmHg diastolic it is recommended to administer 10 mg labetalol intravenous within 1-2 minutes. This should be repeated every 10-20 minutes until blood pressure is below 180 mmHg systolic or below 105 mmHg diastolic. 150 mg labetalol is the maximum doses in 24 hours. During this treatment blood pressure should be measured every 15 minutes. If the blood pressure does not respond to labetalol, iv nitroprusside 0,5-10 μg/kg/minute should be added, with continuous blood pressure monitoring. In case the diastolic blood pressure is above 140 mmHg nitroprusside should be administered immediately as stated above. Thrombocyte count is not necessary before starting rt-PA treatment unless a patient is known with thrombocytopenia [21]. Deviations from these recommendations are not regarded as protocol violations, but will be registered.

### Concomittant medication and secondary prophylaxis

All medication used before the stroke may be continued, except anticoagulance. Standard secondary prophylaxis is recommended according to the following scheme:

▪ carbasalate calcium 300 mg - once/daily - 24 hours after rt-PA for 14 days

▪ carbasalate calcium 100 mg - once/daily - 14 days after rt-PA

▪ simvastatine 40 mg - once/daily - 0-24 hours after rt-PA

▪ dipyridamole 200 mg - twice/daily - 24 hours after rt-PA

Additional anti-diabetic or antihypertensive medication may be started as regarded appropriate by the treating physician.

### Outcome measures

The primary endpoint is poor functional health at 3 months defined as dependency or death (mRS 3 - 6).

The secondary objectives are:

▪ complications within 48 hours after randomisation including the occurrence of SICH and serious systemic bleeding. SICH is defined as CT-documented haemorrhage and a clinical deterioration leading to 4 or more points increase on the National Institute of Health Stroke Scale (NIHSS) as compared to the best score on the NIHSS since admission. Serious systemic bleeding is defined as a potentially life threatening bleeding which requires immediate medical intervention;

▪ neurological symptoms quantified by the NIHSS 7 - 10 days after randomisation or at discharge if the patient is discharged within 7 days;

▪ survival at 3 months;

▪ disability at 3 months assessed by the AMC Linear Disablility Scale;

▪ functional health at 3 months non-dichotomized (ordinal mRS);

▪ causes of poor outcome.

### Data collection

At baseline following patient characteristics are collected at each participating site: age, sex, ethnicity, medical history, pre-stroke medication, pre-stroke mRS, blood pressure, Glasgow Coma Scale (GCS), National Institutes of Health Stroke Scale (NIHSS), time of symptom onset, rt-PA bolus and (if applicable) trial medication, thrombocyte count and coagulation-International Normalized Ratio. Baseline CT-scans will be collected from participating centres and assessed blindly centrally at the coordinating centre for dens media sign, early ischemic changes and degree of leukoariosis by an independent blinded neuro-radiologist.

At follow-up, neurological deficits are assessed by the NIHSS at 7-10 days or at discharge, if this is before 7 days. Clinical deterioration, defined as a 4 or more points increase on the NIHSS, will be followed by CT-scan and registration as (serious) adverse events including possible cause by each participating site. This CT-scan will be assessed at the coordinating centre as well.

Primary outcome will be assessed by a blind research nurse from the clinical trial office of the coordinating centre, who will score the mRS by telephone using a structured interview. To increase the inter-observer reliability the number of research nurses will be limited to a maximum of three. Disability will be assessed by the same research nurse during the same telephone interview using the Amsterdam Linear Disability Scale [22]. See Additional file 1 for all data collection forms.

In patients with a poor outcome at three months, the Data Collection Committee composed of the investigators of the coordinating centre and the local investigator, judges whether this poor outcome is attributed to the initial ischemic stroke, reported adverse event or other causes.

### Safety reporting

All adverse event reported by the subjects or observed by the treating physicians will be recorded. In case of serious adverse events (SAE), the principal investigator will be notified by email or telephone within 24 hours. The principal investigator subsequently reports SAE to the Data Safety Monitoring Board (DSMB). This is an independent committee of trial experts, who will focus on both safety monitoring and analysis of effectiveness on unblinded data. The DSMB will perform ongoing safety surveillances, especially with regard to the occurrence of serious adverse events in terms of SICH and serious systemic bleeding within 48 hours. The DSMB can recommend the Steering Committee of the ARTIS-Trial to terminate the trial when there is clear and substantial evidence of harm. All SAE will be reported to the central METC according to their requirements as well.

### Trial size

Based on our own experience in the stroke unit cohort and the results of the rt-PA thrombolysis trials [1] and the SITS-MOST registry [23] it is expected that 50% of the patients with an ischemic stroke treated with rt-PA thrombolysis will have a poor outcome (mRS 3-6). We aim to reduce this percentage by 10%, a relative risk reduction of 20%.

A two group X^2 ^test with a 0,05 two-sided significance level will have 80% power to detect the difference between the control group proportion of 0,50 and an experimental group proportion of 0,40 (odds ratio of 0,667) when the sample size in each group is 400 (total trial size 800). With this sample size, a two-sided 95% confidence interval for the difference between the proportions will extend 0,069 from the observed difference in proportions. With this sample size we are also able to statistically detect a minimal effect size (difference between mean scores of both treatment arms divided by the SD of the control group ) of *d *= 0,20 as benchmark for assessing the relative magnitude of score differences on the continuous AMC Linear Disability Scale (ALDS) which is a secondary outcome parameter.

### Statistical analyses

Baseline characteristics will be summarized using descriptive statistics. The main analysis of this trial consists of a single comparison between the trial medication groups of the primary outcome after three months (dichotomized Rankin score). The analysis will be based on the intention-to-treat principle. The effect size will be expressed in a relative risk (RR) estimates and absolute risk reduction (ARR). Additionally the primary outcome will be analyzed using multivariate logistic regression, adjusting (if necessary) for clinically relevant baseline imbalances. The differences between NIHSS, ALDS scores and non-dichotomized mRS will be analyzed using the two-group *t*-test, the Mann-Whitney test, linear regression and ordinal logistic regression, when appropriate. The remaining secondary outcomes will be analyzed using simple 2 × 2 tables and logistic regression. In all analyses, statistical uncertainty will be quantified via 95% confidence intervals.

### Interim analysis

Besides interim analyses on the safety data the DSMB will also perform an unblinded interim analysis on the primary outcome to assess the strength of the efficacy data when half of the patients are enrolled. The DSMB will also check the assumptions for sample size calculations. The analysis will be performed by an independent statistician of the Academic Medical Centre Clinical Research Unit, who is not involved in managing the trial. The DSMB can recommend the Steering Committee of the ARTIS-Trial to

▪ adjust the sample size;

▪ early terminate the study when there is clear and substantial evidence of benefit;

▪ early terminate the study in case the data suggests no benefit or in case accrual rates are too low to provide adequate statistical power for identifying the primary endpoint.

### Predefined subgroup-analysis

With respect to the primary outcome a predefined subgroup-analyses will be performed:

▪ rt-PA treatment < 2 hours versus 2-3 hours versus > 3 hours from symptom onset Effectiveness of IV thrombolysis declines over time from symptom onset probably caused by an increase in clot stability. Regarding clot dissolution and reocclusion the beneficial effect of adding antiplatelet therapy might therefore be different over time. The risk of bleeding can also change over time [2].

▪ trial medication within 1 hour versus between 1-1.5 hours from rt-PA bolus. Reocclusion occurs at a median time of 65 minutes after start of rt-PA treatment. Administration of Aspegic in the first hour after the start of rt-PA treatment is therefore expected to result in better outcome [6].

▪ based on ethnicity differences: whites versus blacks, whites versus Hindu's, whites versus blacks and Hindu's, Hindu's versus the other ethnic groups. Previous studies on thrombolytic therapy in acute myocardial infarction suggest that racial differences do exist with an increased thrombolytic effect in blacks accompanied by an increased risk of bleeding complications. The beneficial or detrimental effect of the addition of antiplatelet therapy to IV rt-Pa might therefore differ among different ethnic groups [24-26].

Subgroup analyses consist will consist of a simple comparison of these different groups on primary and secondary outcome measures.

### Ethical considerations

The ARTIS study will be conducted according to the principles of the Declaration of Helsinki (version of 2004) and in accordance with the Medical Research Involving Human Subjects Act (WMO) and other guidelines, regulations and Acts. The Medical Ethics Committee of the Academic Medical Centre approved the protocol before start of the trial. Data management, monitoring and reporting of the study will be performed in accordance with the ICH GCP guidelines. Approval by the local medical ethical review board is required for each participating centre before start of inclusion.

The AMC Medical Research BV has insurance, which is in accordance with the legal requirements in The Netherlands (Article 7 WMO and the Measure regarding Compulsory Insurance for Clinical Research in Humans of June 23, 2003). This insurance provides cover for damage to research subjects through injury or death caused by the trial.

### Publication policy

The trial results will be published by the coordinating investigator on behalf of the ARTIS-study group. Members of the ARTIS-study group will then be listed at the end of the article.

## Discussion

We present the protocol of a randomised controlled clinical trial to investigate the efficacy of direct addition of 300 mg aspirin to rt-PA thrombolysis in acute ischemic stroke. In accordance with thrombolysis in myocardial infarction, in which the combination of acute aspirin and thrombolysis improves outcome considerably [12], we hypothesize that immediate platelet inhibition will improve outcome in acute ischemic stoke by enhancing thrombolysis and preventing re-occlusion after initial recanalisation. As far as we know, this is the first clinical trial investigating the efficacy of direct addition of aspirin to intravenous rt-PA for acute ischemic stroke.

A major safety concern in this trial refers to the occurrence of symptomatic intracranial haemorrhage (SICH). Subgroup-analysis of patients receiving APT *within *24 hours after rt-PA thrombolysis in the first European Cooperative Acute Stroke Study (ECASS-I) showed a slight trend towards increased mortality from all causes (including SICH). This risk is now explained by the higher rt-PA dose (1.1 mg/kg) used in this trial since there was no increased risk in ECASS-II where the currently standard dosage of 0.9 mg/kg rt-PA was used [1].

Several cohort studies could not find a significant association between pre-treatment with antiplatelet agents and SICH [27-30]. Other prospective observational studies observed even a net benefit in favourable outcome after 3 months in patients using antiplatelet drugs prior to rt-PA thrombolysis, despite a strong relationship between this antiplatelet therapy and SICH [18,19]. Recent results from the large SITS-MOST registry of more than 6,000 stroke patients treated with intravenous rt-PA confirmed the increased risk of SICH in patients with antiplatelet pre-treatment [31]. Previous use of aspirin had an odds ratio of 1.58 (95% CI 1.04 - 2.39) of SICH per SITS-MOST definition, a remote parenchymal haemorrhage type 2 on the 22 - 36 hours follow-up imaging scans after the start of thrombolysis treatment. The clinical relevance of these SICH remains to be determined since independency and mortality within 3 months were not associated with previous aspirin use in this registry. Although prior antiplatelet therapy is a contra-indication in this protocol, we are aware of the possible increased risk of SICH due to the combination of rt-PA and aspirin. Therefore, the DSMB will continuously monitor serious adverse events in relation to efficacy outcome measures.

ARTIS is a randomised controlled trial investigating the efficacy of the acute addition of aspirin to intravenous rt-PA thrombolysis in patients with acute ischemic stroke. ARTIS will answer a highly relevant question in acute stroke care by means of a simple adjustment of current antiplatelet regimen with regard to rt-PA thrombolysis. A condensed version of the protocol has been published in Cerebrovascular Diseases [32].

The ARTIS-Trial has started at the end of 2008. Thirty-seven centres are actively randomizing patients. As of May 10^th ^2010, 361 of the 800 patients have been included so far. This trial is set up in the Netherlands. However, other centres - also from foreign countries - experienced in thrombolysis are invited to participate as well. The principle investigator can be contacted by e-mail.

## Abbreviations

APT: antiplatelet therapy; ASA: acetylsalicylic acid; ICH: intracranial haemorrhage; mRS: modified Rankin Scale; NIHSS: National Institute of Health Stroke Scale; rt-PA: recombinant tissue plasminogen activator, SICH: symptomatic intracranial haemorrhage.

## Competing interests

The authors declare that they have no competing interests.

## Authors' contributions

SMZ wrote all drafts and final manuscript, is concerned with patient recruitment and data management. YBR is the principal investigator and conceived the study, designed the protocol, applied for financial support and commented on all drafts and final manuscript. RV, JS and RJH helped conceive the study and design the protocol and commented on final manuscript.

All authors read and approved the final manuscript.

## Supplementary Material

Additional file 1 Data collection forms. Click here for file
