# Supplementary material for: A randomised controlled trial of antiplatelet therapy in combination with Rt-PA thrombolysis in ischemic stroke: rationale and design of the ARTIS-Trial
Source: Trials. 2010 May 12;11:51. doi: 10.1186/1745-6215-11-51 (PMC2886039; doi:10.1186/1745-6215-11-51)
Supplement: Additional file 1 — Data collection forms. [file 1745-6215-11-51-S1.PDF]

DATA COLLECTION FORMS PARTICIPATING SITES

VISIT 1: BASELINE

	**Inclusion criteria <u>known at time of randomisation</u>**	**Yes**	**No**
1.	The patient received rt-PA thrombolysis treatment for an ischemic stroke	☐	☐
2.	The patient is aged ≥ 18 years	☐	☐
3.	Written informed consent is obtained	☐	☐

	**Exclusion criteria <u>known at time of randomisation</u>**	**Yes**	**No**
1.	Known antiplatelet therapy in the previous 5 days<br>(in case of uncertainty the patient may be included)	☐	☐
2.	Known thrombocytopenia (thrombocyte count ≤ 100 * 10E9/l	☐	☐
3.	Contra-indication for aspirin	☐	☐
4.	Known anticoagulant therapy in the previous 5 days	☐	☐
5.	Known legal incompetence of the patient prior to stroke	☐	☐

Demography

Date of birth: |__|__| |__|__|__| |__|__|__|__|
d d m m m y y y y

Sex:
- ☐ Male
- ☐ Female

Ethnicity:
- ☐ White (e.g. European, North African)
- ☐ Black (e.g. African, African-American)
- ☐ Hindu (Surinaams-Hindoestaans)
- ☐ Chinese
- ☐ Other
- ☐ Unknown

**Medical history**		Yes	No
1.	Cerebral ischemia (TIA or infarction)	☐	☐
2.	Intracerebral haemorrhage	☐	☐
3.	Hypertension	☐	☐
4.	Diabetes Mellitus	☐	☐
5.	Hypercholesterolemia	☐	☐
6.	Ischemic heart disease	☐	☐
7.	Peripheral arterial disease	☐	☐
8.	Coagulation disorder	☐	☐

**Relevant medication**		Yes	No
1.	**Dihydropyridinen Calciumantagonists:**<br>Amlodipine (Norvasc) Barnidipine (Cyress) Felodipine (Plendil)<br>Isradipine (Lomir) Iacidipine (Motens) Iercanidipine (Lerdip)<br>Nicardipine (Cardene) Nifedipine (Adalat) Nimodipine (Nimotop)<br>Nitrendipine (Baypress)	☐	☐
2.	**Insulin**	☐	☐
3.	**Statin**	☐	☐
4.	**Anticoagulant therapy** (unknown at time of randomisation)<br>Acenocoumarol (Sintrom) Fenprocoumon (Marcoumar)	☐	☐
5.	**Antiplatelet therapy** (unknown at time of randomisation):<br>Acetylsalicylic Acid (Aspirine) Carbasalatecalcium (Ascal)<br>Dipyridamole (Persantin) Clopidogrel (Plavix)	☐	☐

Modified Rankin Scale score *prior* to stroke

Any disability at all prior to stroke (let op: dus vóór beroerte) |__| Yes |__| No

If yes, only one of the following applies, choose one

1. No significant disability despite symptoms; able to carry out all usual duties and activities ☐
2. Slight disability; unable to carry out all previous activities, but able to look after own affairs without assistance ☐
3. Moderate disability; requiring some help, but able to walk without assistance ☐
4. Moderately severe disability; unable to walk without assistance and unable to attend to own bodily needs without assistance ☐
5. Severe disability; bedridden, incontinent and requiring constant nursing care and attention ☐

Vital signs: blood pressure (mmHg)

Systolic |__|__|__| mmHg

Diastolic |__|__|__| mmHg

Physical examination at baseline

Glasgow Coma Scale:

Eye opening (E) |__|	Motor response (M) |__|	Verbal response (V) |__|
1 = none	1 = none	1 = none
2 = to pain	2 = extension	2 = incomprehensible
3 = to speech	3 = flexor response	3 = inappropriate
4 = spontaneous	4 = withdrawal	4 = confused
	5 = localizes pain	5 = oriented
	6 = obeys commands	A = aphasia
		T = intubated

NIH-Stroke Scale at baseline. Every item must be scored

1a.	Level of consciousness (LOC)	0 = Alert, keenly responsive<br>1 = Not alert, but arousable by minor stimulation<br>2 = Not alert, requires repeated stimulation to attend<br>3 = No response, other then reflexive posturing	__
1b.	LOC questions	0 = Answers both questions correctly<br>1 = Answers one question correctly<br>2 = Answers neither question correctly	__
1c.	LOC commands	0 = Performs both tasks correctly<br>1 = Performs one task correctly<br>2 = Performs neither task correctly	__

2.	Best gaze	0 = Normal<br>1 = Partial gaze palsy<br>2 = Forced deviation	__
3.	Visual fields	0 = No visual loss<br>1 = Partial hemianopia<br>2 = Complete hemianopia<br>3 = Bilateral hemianopia (including (cortical) blindness)	__
4.	Facial palsy	0 = Normal symmetrical movements<br>1 = Minor paralysis (flattened nasolabial fold)<br>2 = Partial paralysis (total lower face paralysis)<br>3 = Complete paralysis (upper and lower face)	__
5.	Motor arm	0 = No drift<br>1 = Drift, drifts down before full 10 sec, does not hit bed<br>2 = Some effort against gravity, hits bed before 10 sec<br>3 = No effort against gravity, arm falls<br>4 = No movement<br>UN = Amputation or joint fusion	L __<br>R __
6.	Motor leg	0 = No drift<br>1 = Drift, drifts down before full 5 sec, does not hit bed<br>2 = Some effort against gravity, hits bed before 5 sec<br>3 = No effort against gravity, leg falls<br>4 = No movement<br>UN = Amputation or joint fusion	L __<br>R __
7.	Limb ataxia	0 = Absent<br>1 = Present in one limb<br>2 = Present in two limbs<br>UN = Amputation or joint fusion	__
8.	Sensory	0 = Normal<br>1 = Mild-to-moderate sensory loss<br>2 = Severe to total sensory loss	__
9.	Best language	0 = Normal<br>1 = Mild-to-moderate aphasia<br>2 = Severe aphasia<br>3 = Mute, global aphasia	__
10.	Dysarthria	0 = Normal<br>1 = Mild-to-moderate dysarthria<br>2 = Severe dysarthria<br>UN = Intubated, other physical barrier	__
11.	Extinction and inattention	0 = No abnormality<br>1 = Visual, tactile, auditory, spatial or personal inattention<br>2 = Profound hemi-inattention or more modalities	__
	Total score		__

Radiology

Head CT-scan

Performed: Yes |__| No |__|

All CT-scans will be evaluated blindly at the coordinating center.

→ Please send a CD-Rom with all head CT-scans to:

Academisch Medisch Centrum
Klinisch OnderzoeksBureau Neurologie
Locatie H2-233
Postbus 22660
1100 DD Amsterdam

Laboratory tests at baseline

Platelet count: |__|__|__| x10E9/L Not available |__|

INR: |__|.|__|__| Not available |__|

Time frame symptom onset and rt-PA treatment

Date of symptom onset |__|__| |__|__|__| |__|__|__|__|
d d m m m y y y y

Time of symptom onset |__|__|:|__|__|
H r min

Date of rt-PA bolus administration |__|__| |__|__|__| |__|__|__|__|
d d m m m y y y y

Time of rt-PA administration |__|__|:|__|__|
H r min

Randomisation

Treatment allocation (ALEA) : |__| Aspégic 300mg |__| No Aspégic

Did patient receive Aspégic 300mg iv? |__| Yes |__| No

Date of Aspégic administration: |__|__| |__|__|__| |__|__|__|__|
d d m m m y y y y

Time of Aspégic administration: |__|__|:|__|__|
H r min

VISIT 2: early follow-up (7 – 10 days or at discharge if discharge < 7 days)

Date of examination: |__|__| |__|__|__| |__|__|__|__|
d d m m m y y y y

**NIH Stroke Scale Score at 7-10 days. Every item must be scored!**			
1a.	Level of consciousness (LOC)	0 = Alert, keenly responsive<br>1 = Not alert, but arousable by minor stimulation<br>2 = Not alert, requires repeated stimulation to attend<br>3 = No response, other then reflexive posturing	__
1b.	LOC questions	0 = Answers both questions correctly<br>1 = Answers one question correctly<br>2 = Answers neither question correctly	__
1c.	LOC commands	0 = Performs both tasks correctly<br>1 = Performs one task correctly<br>2 = Performs neither task correctly	__
2.	Best gaze	0 = Normal<br>1 = Partial gaze palsy<br>2 = Forced deviation	__
3.	Visual fields	0 = No visual loss<br>1 = Partial hemianopia<br>2 = Complete hemianopia<br>3 = Bilateral hemianopia (including (cortical) blindness)	__
4.	Facial palsy	0 = Normal symmetrical movements<br>1 = Minor paralysis (flattened nasolabial fold)<br>2 = Partial paralysis (total lower face paralysis)<br>3 = Complete paralysis (upper and lower face)	__
5.	Motor arm	0 = No drift<br>1 = Drift, drifts down before full 10 sec, does not hit bed<br>2 = Some effort against gravity, hits bed before 10 sec<br>3 = No effort against gravity, arm falls<br>4 = No movement<br>UN = Amputation or joint fusion	L __<br>R __
6.	Motor leg	0 = No drift<br>1 = Drift, drifts down before full 5 sec, does not hit bed<br>2 = Some effort against gravity, hits bed before 5 sec<br>3 = No effort against gravity, leg falls<br>4 = No movement<br>UN = Amputation or joint fusion	L __<br>R __
7.	Limb ataxia	0 = Absent<br>1 = Present in one limb<br>2 = Present in two limbs<br>UN = Amputation or joint fusion	__

8.	Sensory	0 = Normal<br>1 = Mild-to-moderate sensory loss<br>2 = Severe to total sensory loss	__
9.	Best language	0 = Normal<br>1 = Mild-to-moderate aphasia<br>2 = Severe aphasia<br>3 = Mute, global aphasia	__
10.	Dysarthria	0 = Normal<br>1 = Mild-to-moderate dysarthria<br>2 = Severe dysarthria<br>UN = Intubated, other physical barrier	__
11.	Extinction and inattention	0 = No abnormality<br>1 = Visual, tactile, auditory, spatial or personal inattention<br>2 = Profound hemi-inattention or more modalities	__
	Total score		__

Visit 3: Discharge

Date of discharge: |__|__| |__|__|__| |__|__|__|__|
d d m m m y y y y

Destination after discharge:
- ☐ Home
- ☐ Rehabilitation facility
  *please specify name: essential for follow-up*________________________________
- ☐ Nursing home
  *please specify name: essential for follow-up*________________________________
- ☐ Other hospital
  *please specify name: essential for follow-up*________________________________
- ☐ Patient has died
- ☐ Unknown

Were there episodes of clinical deterioration or any other (serious) adverse events - including death - during admission? Yes |__| No |__| *If yes please fill out the "(serious) adverse event form"*

Statement local investigator

Hereby I declare that the pages of Visit 1 - 3 have been checked for completeness and accuracy.

Name researcher: ________________________________

Signature researcher: ________________________________

Date: |__|__| |__|__|__| |__|__|__|__|
d d m m m y y y y

Please enter data in Oracle Database at https://oc.amc.nl/artis/index.htm OR send copy or fax completed CRF to: Academisch Medisch Centrum KOB Neurologie, H2-233 , Postbus 22660, 1100 DD Amsterdam, faxnummer: 020-5669290

Patient Identification Number: |__|__|-|__|__|__|

(Serious) Adverse Event Number: |__|__|

(SERIOUS) ADVERSE EVENTS FORMS PARTICIPATING SITES

(SERIOUS) ADVERSE EVENT FORM

Investigator identification

Name: ______________________

Centre: ______________________

Phone number: ______________________

Date of (S)AE: |__|__| |__|__|__| |__|__|__|__|
d d m m m y y y y

Time of (S)AE: |__|__|:|__|__| Unknown |__|
H r min

Is the AE one or more of the following:

1. CT-documented haemorrhage with ↑NIHSS score ≥ 4 compared to best NIHSS since admission — Yes |__| No |__|

2. potentially life-threatening systemic bleeding which requires immediate medical intervention — Yes |__| No |__|

3. death — Yes |__| No |__|

4. other life threatening situation (at time of event) — Yes |__| No |__|

5. AE leading to prolonged hospitalisation — Yes |__| No |__|

6. AE leading to persistent or significant disability — Yes |__| No |__|

If any answer is yes, this is a serious adverse event (SAE)

If SAE, please enter data < 24 hours in Oracle Database at https://oc.amc.nl/artis/index.htm or send e-mail to artis@amc.nl and fax (serious) adverse event form to: 020-5669290

(Serious) Adverse Event Number: |__|__|

Physical examination

Glasgow Coma Scale
Last recorded GCS score **before** (S)AE:

Glasgow Coma Scale:

Eye opening (E) |__|	Motor response (M) |__|	Verbal response (V) |__|
1 = none	1 = none	1 = none
2 = to pain	2 = extension	2 = incomprehensible
3 = to speech	3 = flexor response	3 = inappropriate
4 = spontaneous	4 = withdrawal	4 = confused
	5 = localizes pain	5 = oriented
	6 = obeys commands	A = aphasia
		T = intubated

GCS score **at the time of** (S)AE:

Glasgow Coma Scale:

Eye opening (E) |__|	Motor response (M) |__|	Verbal response (V) |__|
1 = none	1 = none	1 = none
2 = to pain	2 = extension	2 = incomprehensible
3 = to speech	3 = flexor response	3 = inappropriate
4 = spontaneous	4 = withdrawal	4 = confused
	5 = localizes pain	5 = oriented
	6 = obeys commands	A = aphasia
		T = intubated

National Institutes of Health Stroke Scale (NIHSS)

What is the NIHSS score increase **at the time of** the (S)AE ?

- ☐ < 2 points
- ☐ ≥ 2 points but < 4 points
- ☐ ≥ 4 points

Radiology

Head CT-scan

Performed: Yes |__| No |__|

All CT-scans will be evaluated blindly at the coordinating center.

→ Please send a CD-Rom with all head CT-scans to:

Academisch Medisch Centrum
Klinisch OnderzoeksBureau Neurologie
H2-233, tnv Y. Roos
Postbus 22660
1100 DD Amsterdam

(Serious) Adverse Event Number: |__|__|

Severity

Severity of (S)AE:
- ☐ mild
- ☐ moderate
- ☐ severe

Presumed cause of (S)AE

Presumed predominant cause of (S)AE **(choose one)**

Haemorrhage
- ☐ Any haemorrhagic transformation in infarct region
- ☐ Any intracerebral haemorrhage in other region
- ☐ Any systemic haemorrhage, please specify location ....

Thrombo-embolic
- ☐ Progressive ischemic stroke
- ☐ New infarction in region of initial stroke
- ☐ New infarction in other region

Other possible causes
- ☐ Allergic reaction
- ☐ Pneumonia
- ☐ Urine tract infection
- ☐ Epileptic seizure
- ☐ Other: ....

(S)AE related to the Aspégic treatment:

- ☐ Not applicable (patient randomised for standard care)
- ☐ Unlikely
- ☐ Possible
- ☐ Probably
- ☐ Definite

Patient Identification Number: |__|__|-|__|__|__|

(Serious) Adverse Event Number: |__|__|

Treatment

Did the (S)AE require intervention? Yes |__| No |__|

If yes, please specify: ....

Outcome

If outcome is unknown at this moment, please continue this (Serious) Adverse Event form and fill in later

Outcome after (S)AE:

- ☐ recovered
- ☐ recovering
- ☐ recovered with sequelae
- ☐ not recovered
- ☐ fatal

Date of this outcome |__|__| |__|__|__| |__|__|__|__|
d d m m m y y y y

Statement local investigator

Hereby I declare that the pages of facultative form (Serious) Adverse Event have been checked for completeness and accuracy.

Name researcher: ______________________________

Signature researcher: ______________________________

Date: |__|__| |__|__|__| |__|__|__|__|
d d m m m y y y y

SAE notification should be within 24 hours
Please enter data in Oracle Database at https://oc.amc.nl/artis/index.htm OR
send copy or fax completed (S)AE form to: Academisch Medisch Centrum
H2-233 Klinisch OnderzoeksBureau, Postbus 22660, 1100 DD Amsterdam, fax: 020-5669290

DATA COLLECTION FORMS COORDINATING CENTER
(FOLLOW-UP & RADIOLOGY)

THREE MONTHS FOLLOW-UP

Follow-up interview performed: Yes |__| No |__|

If no, why not:
- ☐ patient (or relative / caregiver) cannot be reached (lost to follow-up)
- ☐ patient refuses
- ☐ patient has deceased

If yes, the person interviewed was:
- ☐ patient
- ☐ relative / caregiver

Date of interview |__|__| |__|__|__| |__|__|__|__|
d d m m m y y y y

Modified Rankin Scale score

Did the patient have any symptoms left Yes |__| No |__|

If yes, tick one of the boxes below; only one answer possible.

1. No significant disability despite symptoms; able to carry out all usual duties and activities ☐
2. Slight disability; unable to carry out all previous activities, but able to look after own affairs without assistance ☐
3. Moderate disability; requiring some help, but able to walk without assistance ☐
4. Moderately severe disability; unable to walk without assistance and unable to attend to own bodily needs without assistance ☐
5. Severe disability; bedridden, incontinent and requiring constant nursing care and attention ☐
6. Dead ☐

Comments: ....

....

....

AMC Linear Disability Scale score

	ALDS<br><br>Are you able to…	yes	yes with difficulty	no	un-known
1	…ride a bike for at least 2 hours?	☐	☐	☐	☐
2	...carry a shopping bag upstairs?	☐	☐	☐	☐
3	…fetch groseries for 3-4 days?	☐	☐	☐	☐
4	…travel by local bus or tram?	☐	☐	☐	☐
5	...walk for more than 15 minutes?	☐	☐	☐	☐
6	…carry a tray?	☐	☐	☐	☐
7	…to go shopping for clothes?	☐	☐	☐	☐
8	…cut your toe nails?	☐	☐	☐	☐
9	…go to a party?	☐	☐	☐	☐
10	…hang out and take in a load for washing?	☐	☐	☐	☐
11	…vacuum without moving any furniture?	☐	☐	☐	☐
12	…move a bed or a table?	☐	☐	☐	☐
13	….reach into a high cupboard?	☐	☐	☐	☐
14	…walk up a flight of stairs?	☐	☐	☐	☐
15	…write a letter?	☐	☐	☐	☐
16	….cross the road?	☐	☐	☐	☐
17	…have a shower and wash your hair?	☐	☐	☐	☐
18	…put on and take off lace-up shoes?	☐	☐	☐	☐
19	…cut your finger nails?	☐	☐	☐	☐
20	…pick something up from the floor?	☐	☐	☐	☐
21	…read a newspaper?	☐	☐	☐	☐
22	…clear the table after a meal?	☐	☐	☐	☐
23	…peel and core an apple?	☐	☐	☐	☐
24	…prepare breakfast or lunch?	☐	☐	☐	☐
25	…eat a meal at the table?	☐	☐	☐	☐

	ALDS	yes	yes with difficulty	no	un-known
26	…put on/take off socks and slip on shoes?	☐	☐	☐	☐
27	…sit up (from lying) in bed?	☐	☐	☐	☐
28	…answer the telephone?	☐	☐	☐	☐
29	…make coffee or tea?	☐	☐	☐	☐
30	…pul long trousers on?	☐	☐	☐	☐
31	…si ton the edge of a bed from lying down?	☐	☐	☐	☐
32	…wash and dry your lower body?	☐	☐	☐	☐
33	…wash and dry your face and hands?	☐	☐	☐	☐
34	…go to the toilet?	☐	☐	☐	☐
35	…put on and take off a T-shirt?	☐	☐	☐	☐

Was treatment allocation recalled?

☐ Yes, Aspégic treatment

☐ Yes, standard treatment

☐ No

Statement investigator coordinating centre

Hereby I declare that the pages of 3 months follow-up have been checked for completeness and accuracy.

Name researcher: ________________________________

Signature researcher: ________________________________

Date: |__|__| |__|__|__| |__|__|__|__|
d d m m m y y y y

RADIOLOGY

Baseline head CT-scan

Date of radiology report: |__|__| |__|__|__| |__|__|__|__|
d d m m m y y y y

Affected brain side: Left |__| Right |__| Not determined |__|

Any early ischemic changes (EIC) present? Yes |__| No |__|

If EIC, please location: Left |__| Right |__|
Supratentorial |__| Infratentorial |__|

ASPECTS-score: 0 ☐ 1 ☐ 2 ☐ 3 ☐ 4 ☐ 5 ☐ 6 ☐ 7 ☐ 8 ☐ 9 ☐ 10 ☐

Estimated volume of EIC in MCA territory > 1/3? Yes |__| No |__|

Marked hyperdense middle cerebral artery sign: Left |__| Right |__| No |__|

Degree of periventricular leukoariosis:

- ☐ no signs of leukoariosis
- ☐ moderate leukoariosis
- ☐ severe leukoariosis

FOLLOW UP head CT-scan

Follow up head CT-scan performed? Yes |__| No |__|

Date of radiology report: |__|__| |__|__|__| |__|__|__|__|
d d m m m y y y y

Date of performing head CT-scan: |__|__| |__|__|__| |__|__|__|__|
d d m m m y y y y

Time of head CT-scan: |__|__|:|__|__|
H r min

Indication for head CT-scan (choose one)
- ☐ Clinical deterioration < 4 points on NIHSS
- ☐ Clincal deterioration ≥ 4 points on NIHSS
- ☐ Unknown
- ☐ Other *(please specify)* ___________________________

CT-findings (one or more of the following):
- ☐ Unchanged compared to baseline head CT-scan
- ☐ Marked hyperdense middle cerebral artery sign
- ☐ Increase in parenchymal hypoattenuation
- ☐ Increase in ischemic edema *without* mass effect
- ☐ Increase in ischemic edema *with* mass effect
- ☐ Intracerebral hemorrhage (ICH)
- ☐ Unknown
- ☐ Other *(please specify)*___________________________

If ICH, please specify type of hemorrhage (choose one)
- ☐ Hemorrhagic infarction type 1
- ☐ Hemorrhagic infarction type 2
- ☐ Parenchymal infarction type 1
- ☐ Parenchymal infarction type 2
- ☐ Remote primary intracerebral hemorrhage type 1
- ☐ Remote primary intracerebral hemorrhage type 2

Do the radiological findings explain the clinical deterioration?

Yes |__| No |__| Uncertain |__| Not applicable |__|

Statement investigator coordinating centre

Hereby I declare that the pages of radiology have been checked for completeness and accuracy.

Name researcher: ________________________________

Signature researcher: ________________________________

Date: |__|__| |__|__|__| |__|__|__|__|
d d m m m y y y y
